# Toward Effective Educational Supervision in Yemen: A Hybrid Fuzzy Delphi and Clustering Analysis of Technical Barriers

**DOI:** 10.12688/f1000research.167498.2

**Published:** 2026-02-09

**Authors:** Riyadh Ghaleb A. Alshameri, Adel A. Nasser, Abdul Hakim Abdullah, Abed Saif Ahmed Alghawli, Amani A. K. Elsayed

**Affiliations:** 1 Department of Education, Sultan Zainal Abidin University, Kuala Terengganu, Terengganu, Malaysia; 2 Department of Information Systems and Computer Science, Sa’adah University, Sa'adah, Sa'adah, Yemen; 3 Department of Artiﬁcial Intelligence, Modern Specialized University, Sana'a, Sana'a, Yemen; 4 Department of Computer Science, College of Sciences and Humanities, Prince Sattam bin Abdulaziz University, Al Kharj, Riyadh Province, Saudi Arabia

**Keywords:** educational supervision; Fuzzy Delphi Method; Yemen; fuzzy set theory; K-means ; clustering; supervisory competencies

## Abstract

**Objectives:**

Comprehensive educational supervision is essential for ensuring quality teaching, fostering professional development, and supporting institutional capacity building. However, its implementation encounters numerous structural, technical, and human resource challenges. This study aimed to identify, validate, rank, and cluster the technical barriers affecting comprehensive educational supervision in Amanat Al Asimah, Yemen. This aligns with national reform goals by offering strategic insights to improve supervisory systems, thereby enhancing teaching quality, institutional performance, and educational resilience in fragile contexts

**Methods:**

This study employed a three-phase mixed-methods approach. Initially, a literature review identified 11 key barriers to effective supervision. These were validated using the Fuzzy Delphi Method (FDM), involving 16 experienced educational supervisors to assess the consensus and suitability of the items. Subsequently, a quantitative survey targeting 370 teachers was conducted to evaluate their perceived severity. Fuzzy set theory was used to aggregate and defuzzify the responses, generating crisp scores for prioritization. Finally, K-means clustering was applied to segment the barriers based on their impacts.

**Results:**

FDM analysis confirmed the validity of all 11 identified barriers, with a domain-level threshold of 0.093 and an average expert consensus of 89.77%, indicating strong agreement. The fuzzy set-based evaluation highlighted three top-priority challenges: weak supervisory competencies, limited ability to develop effective supervisory plans, and poor supervisor-teacher relationships. K-means clustering grouped the barriers into three segments: one high-priority barrier, seven moderate-priority concerns, and three low-priority issues. Notably, weak supervisory competencies emerged as the most critical barrier, isolated in a high-priority cluster.

**Conclusion:**

These findings provide evidence-based guidance for policy and strategic interventions aimed at enhancing the effectiveness of supervision systems in fragile educational settings. The study concludes with recommendations for strengthening supervisory competencies, improving resource allocation, and fostering trust-based supervisor-teacher relationships, thereby contributing to the quality of education and institutional resilience in Yemen.

**Glossary**
FDM: Fuzzy Delphi Method
MCDM: Multi-Criteria Decision-Making.
B1: Teachers’ disinterest in supervisors’ directives
B2: Limited capability of certain educational supervisors in creating effective supervisory plans
B3: Limited capability of certain educational supervisors in creating and implementing effective supervisory training programs
B4: Weak competencies of educational supervisors in supervision
B5: Limited teacher acceptance of modern supervisory methods
B6: Poor relationships between supervisors and teachers
B7: Lack of access to modern technologies
B8: Inadequate financial allocations for supervision activities
B9: Insufficient availability of learning resources in schools
B10: Absence of training and rehabilitation halls
B11: Lack of dedicated offices for educational supervision

## 1. Introduction

The current global shifts in science and education have significantly impacted various educational dimensions, including systems and supervision processes. These transformations have compelled educators to critically examine educational structures and reaffirm the teacher’s role as a central pillar within the system. ^1^ Within this context, educational supervision has emerged as a pivotal component of educational management, fundamental to the broader educational framework that encompasses both teaching and learning. ^2^ Educational supervision serves as a cornerstone for ensuring educational quality and promoting continuous improvement. It plays a vital role in enhancing teacher performance by offering professional guidance, fostering reflective practice, and aligning teaching with curriculum standards. ^3^ Historically limited to classroom observation, the scope of supervision has expanded to include mentorship, collaborative problem-solving, and the development of targeted professional growth plans. ^4^

In today’s dynamic educational landscape—shaped by digital transformation and evolving pedagogical expectations—supervisors are instrumental in helping educators integrate technology, maintain digital ethics, and implement innovative teaching strategies suited to 21st-century learners. ^5^ Beyond instructional support, effective supervision also nurtures teachers’ emotional well-being and professional development, ensuring that education remains inclusive, adaptive, and student-centered. ^6^ Modern supervisory models now emphasize collaborative learning environments and constructive feedback mechanisms, thereby cultivating a culture of continuous improvement. ^7^ As Okafor et al. note, effective supervision not only enhances instructional practices but also strengthens institutional effectiveness by fostering an environment conducive to both teacher and student success. ^8^ Given its multifaceted nature, educational supervision continues to evolve—requiring ongoing research and innovation to meet the complex demands of contemporary education.

The success of educational supervision hinges on several critical indicators. Supervisors must offer clear directives and align instructional practices with institutional objectives. ^9^ Emphasis should be placed on implementing quality assurance mechanisms that align with global standards while addressing local educational contexts. ^9^ Continuous professional development is essential in fostering a culture that supports quality teaching and lifelong learning. ^10, 11^

Moreover, supervision becomes most effective when teachers actively engage with feedback, thereby promoting a culture of accountability and improvement. ^12^ Effective planning is equally important—supervisors must ensure that their approach is strategic, goal-oriented, and systematic. ^13, 14^ Competent supervisors are characterized by strong instructional leadership, effective communication, accurate observation, and constructive feedback delivery. ^15, 16^ Building trust-based professional relationships between supervisors and teachers further enhances collaboration and facilitates open communication. ^17, 18^ Additionally, the integration of modern technology and the availability of financial and learning resources significantly contribute to the success of supervision practices. ^19^ These factors collectively support a dynamic, responsive, and impactful supervision system.

Ultimately, educational supervision focuses on actively involving teachers in the learning environment to enhance teaching methods and improve student achievement. It plays a central role in overseeing the educational process by integrating instructional activities and continuously evaluating institutional outcomes. ^2, 20^ Through identifying challenges and working collaboratively to resolve them, supervision ensures the implementation of appropriate actions that drive educational advancement.

Numerous studies indicate that educational supervision, in its current state, faces significant challenges that hinder its effectiveness. Including inadequate training programs, ^10^ limited planning, ^21^ weak supervisory competencies, ^15^ low teacher acceptance of modern methods, ^22^ strained relationships, ^18^ technological gaps, ^19^ and insufficient financial, infrastructural, and learning resources. ^2, 23^ To overcome these barriers, studies recommend structured professional development, strategic planning, competence-based supervisor selection, ^16^ fostering collaborative cultures, ^24^ integrating digital tools, ^25^ securing adequate funding, ^26^ and providing supportive environments to strengthen supervision’s impact.

Comprehensive educational supervision is a fundamental approach employed in schools to assess and respond to their challenges, needs, and educational and social realities. It involves the continuous presence of educational supervisors within school environments to gain firsthand insights into the teaching and learning processes both inside and outside the classroom. Through this approach, supervisors are able to diagnose the real conditions of the educational system—including the roles of teachers, learners, school leadership, and the broader learning environment.

However, in Yemen, the ability to implement effective supervision has been severely undermined by ongoing conflicts and political instability. The education system, which once showed progress with enrollment rates increasing from 71.3% to 97.5% between 1999 and 2013, has since experienced a catastrophic reversal. ^27^ Recent data reveal that over 2,400 schools are now damaged or unfit for educational use, ^28^ while the population of out-of-school children has reached approximately 3.7 million. ^29^ This systemic collapse is exacerbated by a near-total breakdown in teacher support systems, with the majority of educators unpaid for years. Furthermore, reports of near-universal learning poverty among children indicate a precipitous decline in education quality.

These structural disruptions have weakened the foundation of educational supervision. Furthermore, the quality of education has declined significantly, with reports of curriculum manipulation for ideological purposes and widespread issues such as the absence of teacher training and salary payments. ^30^ In response to these profound challenges, Yemen’s National Vision for Building the Modern Yemeni State and the national research priorities emphasize the urgent need for comprehensive education reform.

In line with these priorities, this study aims to analyze the technical challenges facing educational supervision in schools located in Amanat Al Asimah and explore viable strategies to improve the efficiency of educational processes. The focus is on supporting national goals by modernizing supervisory practices and ensuring they contribute meaningfully to sustainable development and institutional capacity building. The study seeks to identify, rank, and cluster these challenges in schools within Amanat Al Asimah, Yemen, while exploring viable strategies to enhance the efficiency of educational supervision processes.

The significance of this study lies in its alignment with Yemen’s National Vision for Building a Modern Yemeni State and national research priorities. It contributes to modernizing supervisory practices in a conflict-affected context, supports sustainable development, and enhances institutional capacity in Yemen’s education sector. Additionally, it provides insights into addressing teacher-related challenges and improving the quality of education.

Methodologically, the study employs a mixed-methods approach that includes a literature review to identify key barriers to effective supervision, the Fuzzy Delphi Method to validate and prioritize these barriers, a quantitative survey of teachers to assess their perceptions of supervisory challenges, and fuzzy set theory and K-means clustering analysis to categorize and prioritize the identified barriers.

The findings of this study could inform targeted policy interventions to enhance supervisory effectiveness, guide resource allocation for improving educational supervision, support capacity-building initiatives for supervisors, and contribute to developing a responsive supervision framework aligned with national development objectives.

## 2. Methods

Multi-Criteria Decision-Making (MCDM) methods have emerged as vital analytical tools in a wide range of fields, including health, sustainability, tourism, healthcare, engineering, education and financial risk assessment. ^31– 41^ The integration of MCDM methods with fuzzy logic and clustering techniques provides a robust framework for addressing complex decisions under uncertainty. ^31– 35^ Fuzzy-based MCDM approaches effectively capture expert ambiguity and contextual nuances, making them particularly suitable for fields such as health, education, and sustainability. ^37, 40– 42^ The Fuzzy Delphi Method (FDM), enhanced by fuzzy set theory, improves expert consensus and facilitates clearer selection and prioritization of variables. ^37, 40, 43, 44^

Meanwhile, machine learning (ML) has significantly advanced real-world decision-making through predictive modeling, risk analysis, and pattern recognition. ^45– 50^ Its applications span various domains, including healthcare, ^45^ big data analytics, ^46^ manufacturing quality control, ^47^ and infrastructure forecasting. ^48^ ML also supports academic evaluation through web mining, ^49^ data mining, ^50^ and Industry 4.0 applications. ^51^

Recent research increasingly integrates MCDM, fuzzy logic, and unsupervised ML techniques—particularly K-means clustering—to enhance decision-making by segmenting barriers and identifying strategic priorities. ^31– 35^ This hybrid approach has been successfully applied in health security, ^31– 35^ ecotourism, ^41^ urban resilience assessment, ^52^ and education, ^42, 53^ offering scalable, context-sensitive solutions to complex policy and planning challenges. This study is grounded in a two-tiered hybrid analytical framework that integrates methodological approaches at the stages of barrier validation and data-driven prioritization. The framework sequentially combines the following:

• A mixed qualitative-quantitative technique, the Fuzzy Delphi Method (FDM), was used for expert-driven identification, validation, and preliminary ranking of barriers.

In this core phase, the Fuzzy Delphi Method (FDM) served as the foundational mixed-method tool. ^54^ Its qualitative stage involved the identification of preliminary barriers through literature analysis and the collection of linguistic judgments from a panel of 16 educational experts. ^44, 55^ Its quantitative stage converted these qualitative judgments into fuzzy numbers, applied mathematical aggregation and defuzzification using Fuzzy Set Theory, and employed statistical consensus thresholds to validate and preliminarily rank the final set of 11 technical barriers (B1–B11). ^44, 55^ Within the FDM, Fuzzy Set Theory functions as the core computational mechanism, enabling the transformation of expert input into quantifiable data prior to consensus evaluation.

• Quantitative techniques—Fuzzy Set Theory and K-means Clustering—for data-driven ranking and segmentation of barriers based on teacher perceptions.

In the second phase (Quantitative Extension and Synthesis), the validated barriers were then analyzed using quantitative methods. Fuzzy Set Theory was applied independently a second time to the teacher survey data (n=370) to aggregate responses and defuzzify them into crisp scores for ranking barrier severity. ^36, 40, 41^ These scores served as inputs for K-means clustering, an unsupervised machine learning technique used to segment barriers into distinct priority groups based on their perceived impact patterns. ^31– 34^ This phase translated the validated barriers into an empirically grounded, prioritized framework.

• Synthesis of findings and development of evidence-based strategic recommendations.

**Figure 1.**
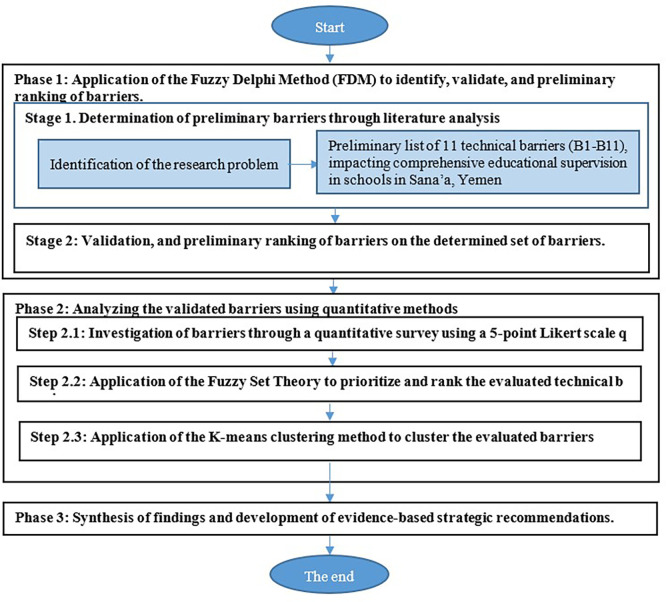
Sequential mixed-methods research design.

This layered hybrid approach—FDM (Mixed) with Fuzzy Ranking & Clustering (Quantitative)—ensures that the study’s outcomes are both contextually validated by local expertise and empirically robust. The methodological flow is shown in Figure 1.

The following subsections detail the procedural stages, with each stage clearly outlined and supported by a rationale for the chosen methodology.

### 2.1 Phase 1 - application of the Fuzzy Delphi Method (FDM) to identify, validate, and preliminary ranking of barriers

The Fuzzy Delphi Method (FDM) is a widely used technique for assessing the suitability of research indicators in measuring specific phenomena within defined environments because of its ability to combine expert judgment with the flexibility of fuzzy logic. ^37, 41, 42^ Often, determining the relevance of indicators requires subjective input from domain experts, whose perspectives may vary or be influenced by uncertainties arising from complex contextual conditions. ^55^ FDM addresses this challenge by allowing experts to provide assessments using linguistic terms, which are then converted into fuzzy numbers, enabling the method to handle ambiguity and imprecision more effectively. ^37, 55^ Compared to the traditional Delphi method, ^43^ which often requires multiple rounds of consultation to reach a consensus and may lead to misinterpretation or loss of expert interest due to repetitive rounds, FDM enhances efficiency by minimizing the number of iterations needed. It also reduces the risk of bias introduced through forced modifications of expert opinions and lowers the overall cost and time required for data collection. ^37, 41^

Additionally, the FDM increases survey response rates, improves the reliability of expert feedback, and effectively resolves issues related to non-consensus cases. This helps make evaluations more consistent and clear, keeping expert opinions genuine while encouraging agreement and realism in finding relevant indicators. The method further provides a systematic approach to filter and validate indicators by applying a consensus threshold, ensuring the retention of only the most relevant and context-appropriate variables. Owing to its adaptability across domains and environments, FDM enables experts to assess indicator suitability based on theoretical soundness and practical relevance to the target context. ^35, 39, 42^ Overall, by merging expert knowledge, fuzzy logic, and a structured consensus mechanism, the Fuzzy Delphi Method (FDM) enhances the robustness, credibility, and contextual relevance of selected indicators, making it particularly valuable in complex evaluation frameworks, such as the assessment of educational barriers in low-income countries and other context-sensitive settings.

In this study, the following FDM procedural stages and steps were implemented ^44, 55^:

Stage 1: Identification of Key Evaluation Barriers through Literature Review

A substantial body of research underscores the significance of educational supervision in enhancing instructional quality, fostering professional development, and aligning teaching practices with institutional and national objectives. However, in fragile contexts such as Yemen, its effectiveness is severely constrained by a well-documented set of interconnected barriers. This review synthesizes the literature into 11 factors (see Table 1).

B1) Teachers’ Disinterest in Supervisory Directives: A key impediment is the widespread lack of interest in and resistance to supervisors’ instructions. This disengagement fundamentally undermines improvement initiatives and reflects broader systemic issues of accountability. ^9, 12, 56, 57^

B2) Supervisors’ Deficient Planning Capacity: The literature consistently identifies the weak capacity of many educational supervisors to formulate effective, goal-oriented supervisory plans. This results in oversight that is inconsistent, fragmented, and reactive rather than systematic. ^13, 14, 58, 59^

B3) Ineffective Training Program Development: The limited ability of supervisory bodies to design and implement robust training programs is closely linked. This deficiency hinders efforts to equip teachers with contemporary skills and align instructional methods with evolving standards. ^10, 12^

B4) Lack of Supervisory Competencies: Many supervisors exhibit critical gaps in core competencies, including instructional leadership, communication, and the delivery of constructive feedback. These deficiencies erode the credibility and professional impact of the supervision process. ^15, 16, 19^

B5) Teacher Resistance to Modern Methods: Reforms aimed at making supervision more participatory and data-driven are often limited by teacher resistance to collaborative methods such as peer feedback and formative evaluation. ^22, 24, 60– 62^

B6) Poor Supervisor-Teacher Relationships: Weak, distant, or mistrustful professional relationships between supervisors and teachers stifle open communication, reduce trust, and lead to superficial or ineffective feedback cycles. ^17, 18, 63, 64^

B7) Lack of Access to Modern Technology: The absence or inadequacy of digital tools—such as learning management systems, video conferencing platforms, and data analytics software—severely limits the efficiency, reach, and adaptability of supervision practices. ^19, 25, 64, 65^

B8) Inadequate Financial Allocation: Chronic underfunding of supervision activities hampers essential functions, including professional development, travel for school visits, logistical support, and the procurement of necessary materials. ^8, 23, 66^

B9) Insufficient School Learning Resources: The scarcity of foundational learning resources in schools, such as functional libraries, science laboratories, computers, and Internet access not only impedes quality teaching but also renders meaningful supervisory assessment nearly impossible. ^19, 67– 69^

B10) Absence of Training and Rehabilitation Halls: The lack of dedicated physical spaces for conducting training workshops and rehabilitation sessions curtails opportunities for organized, continuous professional development for educators. ^2^

B11) Lack of Dedicated Supervision Offices: The absence of specialized offices for educational supervision compromises the coordination, documentation, and institutionalization of supervisory activities, perpetuating an ad hoc and fragmented approach. ^2^

**Table 1.** Key Technical Barriers identified and evaluated.

ID	Barrier
**B1**	Teachers’ disinterest in supervisors’ directives
**B2**	Limited capability of certain educational supervisors in creating effective supervisory plans
**B3**	Limited capability of certain educational supervisors in creating and implementing effective supervisory training programs
**B4**	Weak competencies of educational supervisors in supervision
**B5**	Limited teacher acceptance of modern supervisory methods
**B6**	Poor relationships between supervisors and teachers
**B7**	Lack of access to modern technologies
**B8**	Inadequate financial allocations for supervision activities
**B9**	Insufficient availability of learning resources in schools
**B10**	Absence of training and rehabilitation halls
**B11**	Lack of dedicated offices for educational supervision

Stage 2: Validation, and preliminary ranking of barriers on the determined set of barriers.

• Step 1: Expert Selection and Data Collection

In accordance with FDM guidelines, this study purposively selected a panel of experts to ensure homogeneity, as recommended in previous research. ^41^ A total of 16 educational supervisors were chosen based on their direct involvement in educational supervision and extensive experience—each with over 10 years in the field. A structured questionnaire was used to collect data, consisting of 11 barriers to effective supervision. Fifteen printed questionnaires were distributed and collected manually. Experts were asked to rate the relevance and contextual suitability of each barrier within the Yemeni educational environment using a five-point Likert scale (1 = strongly disagree, 5 = strongly agree). The collected responses (see Table 2) were then converted into fuzzy numbers using the conversion scale proposed by. ^44, 55^( Table 3).

**Table 2.** Experts’ scores using a five-point likert scale.

Expert No	B1	B2	B3	B4	B5	B6	B7	B8	B9	B10	B11
**E1**	4	5	4	5	4	4	5	5	5	4	4
**E2**	4	5	5	5	5	5	5	4	5	3	4
**E3**	4	5	5	5	5	5	4	5	5	4	4
**E4**	4	5	4	5	4	4	5	5	5	4	4
**E5**	4	4	5	4	5	5	5	5	5	3	4
**E6**	5	4	4	4	5	5	4	5	5	4	4
**E7**	4	5	4	5	4	4	5	5	5	4	4
**E8**	4	5	5	5	5	5	5	5	5	4	4
**E9**	5	5	4	5	5	5	5	5	5	4	3
**E10**	4	5	4	5	4	5	5	5	5	4	4
**E11**	4	4	5	4	5	5	5	5	5	4	4
**E12**	5	5	4	5	4	4	4	5	5	4	3
**E13**	4	5	4	5	4	5	5	5	5	3	4
**E14**	4	5	5	5	5	4	5	5	5	4	4
**E15**	4	5	4	5	4	4	5	5	5	4	4
**E16**	4	5	5	5	5	5	5	5	5	4	4

Table displays the raw scores given by 16 educational supervision experts for the 11 identified barriers to effective supervision. Each expert assessed the relevance and contextual suitability of these barriers within the Yemeni educational environment using a five-point Likert scale ranging from 1 (Strongly Disagree) to 5 (Strongly Agree).

**Table 3.** Mapping likert to fuzzy scoring scale.

Likert scale scoring	Linguistic variable	Fuzzy Scale scoring		
		m1	m2	m3
**5**	Highly Agree	0.6	0.8	1
**4**	Agree	0.4	0.6	0.8
**3**	Moderately/Not sure	0.2	0.4	0.6
**2**	Not Agree	0	0.2	0.4
**1**	Highly Not Agree	0	0	0.2

Table details the conversion of the Likert scale ratings into the corresponding triangular fuzzy numbers. Each linguistic variable, from “Highly Not Agree” to “Highly Agree,” is mapped to a triplet (m1, m2, m3) based on the fuzzy scale scoring method adopted from previous studies.

• Step 2: Fuzzy Data Analysis
  ○ Step 2.1: Aggregation of Fuzzy Ratings

In fuzzy set theory, each element is characterized by a pair: the value and its degree of membership in the fuzzy set, defined within the [0,1] interval. A membership function quantifies this degree, with values closer to 1 indicating a stronger inclusion in the fuzzy set. As detailed in Table 3, fuzzy values were used to represent the uncertain expert responses (see Table 4).

**Table 4.** Experts’ scores using a fuzzy rating scale.

Expert No	B1			B2			… .	B11		
	m1	m2	m3	m1	m2	m3	… .	m1	m2	m3
**E1**	0.4	0.6	0.8	0.6	0.6	1	… .	0.4	0.6	0.8
**E2**	0.4	0.6	0.8	0.6	0.8	1	… .	0.4	0.6	0.8
**E3**	0.4	0.6	0.8	0.6	0.8	1	… .	0.4	0.6	0.8
**E4**	0.4	0.6	0.8	0.6	0.8	1	… .	0.4	0.6	0.8
**E5**	0.4	0.6	0.8	0.4	0.6	0.8	… .	0.4	0.6	0.8
**E6**	0.6	0.8	1	0.4	0.6	0.8	… .	0.4	0.6	0.8
**E7**	0.4	0.6	0.8	0.6	0.8	1	… .	0.4	0.6	0.8
**E8**	0.4	0.6	0.8	0.6	0.8	1	… .	0.4	0.6	0.8
**E9**	0.6	0.8	1	0.6	0.8	1	… .	0.2	0.4	0.6
**E10**	0.4	0.6	0.8	0.6	0.8	1	… .	0.4	0.6	0.8
**E11**	0.4	0.6	0.8	0.4	0.6	0.8	… .	0.4	0.6	0.8
**E12**	0.6	0.8	1	0.6	0.8	1	… .	0.2	0.4	0.6
**E13**	0.4	0.6	0.8	0.6	0.8	1	… .	0.4	0.6	0.8
**E14**	0.4	0.6	0.8	0.6	0.8	1	… .	0.4	0.6	0.8
**E15**	0.4	0.6	0.8	0.6	0.8	1	… .	0.4	0.6	0.8
**E16**	0.4	0.6	0.8	0.6	0.8	1	… .	0.4	0.6	0.8
**AFR**	0.438	0.638	0.838	0.563	0.750	0.963	… .	0.375	0.575	0.775
**CS**	0.638			0.758			… .	0.575		

Table shows the fuzzy representation of expert judgment across the 11 identified barriers to effective supervision. Each expert’s rating, initially captured using a five-point Likert scale, was transformed into a corresponding triangular fuzzy number (m1, m2, m3) according to the fuzzy scale defined in Table 2. The table also includes the Aggregated Fuzzy Ratings (AFR) and defuzzified Crisp Scores (CS) for each barrier, calculated using the centroid method.

The average fuzzy rating (AFR) for each variable (barrier z) was then calculated across all n experts using the following formula:

$$\tilde{w_{z}}=\left(a_{z}.b_{z}.c_{z}\right)=\left(\frac{1}{n}\;\sum_{r=1}^{n}a_{\mathrm{rz}}.\frac{1}{n}\;\sum_{r=1}^{n}b_{\mathrm{rz}}.\frac{1}{n}\;\sum_{r=1}^{n}c_{\mathrm{rz}}\right)$$ (1)

○ Step 2.2: Defuzzification

The fuzzy values for each variable were then converted into crisp scores (CS) (See Table 4) using the centroid method (center of gravity), defined as:

$$D_{z}=\frac{\left(a_{z}.b_{z}.c_{z}\right)}{3}\;$$ (2)

• Step 3: Evaluation of Domain Acceptability
  ○ Step 3.1: Distance Calculation

As shown in Table 5, the deviation between each expert’s fuzzy rating and the aggregated fuzzy score was computed using the following formula:

$$D_{\mathrm{rz}}=\sqrt{\frac{{\left(a_{z}-a_{\mathrm{rz}}\right)}^{2}+{\left(b_{z}-b_{\mathrm{rz}}\right)}^{2}+{\left(c_{z}-c_{\mathrm{rz}}\right)}^{2}}{3}}$$ (3)

○ Step 3.2: Threshold Value of Each barrier

**Table 5.** Deviation calculation of expert fuzzy ratings versus aggregated scores.

Expert No	B1	B2	B3	B4	B5	B6	B7	B8	B9	B10	B11
**E1**	0.1	0.2	0.1	0.1	0.2	0.2	0.1	0.0	0.0	0.1	0.0
**E2**	0.1	0.1	0.2	0.1	0.1	0.1	0.1	0.3	0.0	0.2	0.0
**E3**	0.1	0.1	0.2	0.1	0.1	0.1	0.2	0.0	0.0	0.1	0.0
**E4**	0.1	0.1	0.1	0.1	0.2	0.2	0.1	0.0	0.0	0.1	0.0
**E5**	0.1	0.2	0.2	0.2	0.1	0.1	0.1	0.0	0.0	0.2	0.0
**E6**	0.2	0.2	0.1	0.2	0.1	0.1	0.2	0.0	0.0	0.1	0.0
**E7**	0.1	0.1	0.1	0.1	0.2	0.2	0.1	0.0	0.0	0.1	0.0
**E8**	0.1	0.1	0.2	0.1	0.1	0.1	0.1	0.0	0.0	0.1	0.0
**E9**	0.2	0.1	0.1	0.1	0.1	0.1	0.1	0.0	0.0	0.1	0.3
**E10**	0.1	0.1	0.1	0.1	0.2	0.1	0.1	0.0	0.0	0.1	0.0
**E11**	0.1	0.2	0.2	0.2	0.1	0.1	0.1	0.0	0.0	0.1	0.0
**E12**	0.2	0.1	0.1	0.1	0.2	0.2	0.2	0.0	0.0	0.1	0.3
**E13**	0.1	0.1	0.1	0.1	0.2	0.1	0.1	0.0	0.0	0.2	0.0
**E14**	0.1	0.1	0.2	0.1	0.1	0.2	0.1	0.0	0.0	0.1	0.0
**E15**	0.1	0.1	0.1	0.1	0.2	0.2	0.1	0.0	0.0	0.1	0.0
**E16**	0.1	0.1	0.2	0.1	0.1	0.1	0.1	0.0	0.0	0.1	0.0
**Threshold Value**	0.093	0.104	0.150	0.093	0.150	0.143	0.093	0.036	0.000	0.093	0.067
**Domain-Level Threshold**	0.093 < 0.2										

Table presents the distance values calculated between each expert’s fuzzy score and the aggregated fuzzy score for each barrier. These deviations were used to assess the level of agreement between the experts. The final row reports the threshold values for each barrier, while the domain-level threshold confirms the overall acceptability of the evaluation domain, with all threshold values below the 0.2 benchmark.

For each variable, a threshold value was calculated by averaging these distances:

$${\mathrm{Th}}_{z}=\frac{1}{n}\;\sum_{r=1}^{n}D_{\mathrm{rz}}$$ (4)

○ Step 3.3: Domain-Level Threshold

The overall threshold for the entire domain of variables was then determined by averaging the threshold values of all factors:

$${\mathrm{Th}}_{\text{domain}}=\frac{1}{n}\;\sum_{z=1}^{m}{\mathrm{Th}}_{z}$$ (5)

A domain and its variables were considered valid if their threshold values were ≤ 0.2. ^44, 55^ Barriers with threshold values below this criterion were deemed acceptable for further analysis. In this study, As shown in Table 5, the overall domain threshold was calculated as 0.093, while all individual variable threshold values were found to be less than 0.2.

• Step 4: Assessing Expert Consensus

To determine expert consensus on each factor, the number of experts (n) whose ratings were within a fuzzy distance of ≤ 0.2 from the average fuzzy rating ( $E_{z})\;$ was calculated (see Table 6). The percentage of these experts ${\mathrm{EA}}_{z}$ was defined as:

$${\mathrm{EA}}_{z}=\frac{E_{z}\;}{n}\%$$ (6)

**Table 6.** Determining expert consensus through fuzzy distance metrics.

Expert n	B1	B2	B3	B4	B5	B6	B7	B8	B9	B10	B11
** Number of experts, d ≤ 0.2**	13	13	16	13	16	16	13	15	16	13	14
**Percentage of Experts (%), d ≤ 0.2**	81.25%	81.25%	100.00%	81.25%	100.00%	100.00%	81.25%	93.75%	100.00%	81.25%	87.50%
**Average**	89.77%										

Table illustrates the degree of consensus among experts for each barrier based on the fuzzy distance metric. It lists the number and percentage of experts whose ratings deviated from the average fuzzy rating by ≤ 0.2. A consensus threshold of 75% was adopted, and all barriers exceeded this level, indicating a strong agreement within the expert panel.

Consensus was measured by assessing the proportion of experts whose ratings deviated from the group average by less than or equal to 0.2, as indicated in Equation (6). A consensus threshold of 75% was adopted. All 11 barriers met or exceeded this threshold. Subsequently, the final crisp scores were used to rank the barriers in terms of priority, with any factor scoring below 0.4 excluded from further consideration.

• Step 5: Final Evaluation and Variable Selection

After confirming the validity of the evaluation domain and identifying variables with sufficient expert consensus, the final step involved analyzing the crisp scores of the accepted barriers. These scores were used to rank the barriers in order of priority, with any variable scoring below 0.4 excluded from further consideration. However, as presented in the results section, all 11 barriers achieved scores above the threshold and were therefore accepted and retained for further analysis.

### 2.2 Analyzing the validated barriers using quantitative methods

*2.2.1 Investigation of barriers through a quantitative survey using a 5-point Likert scale questionnaire*

To investigate the technical barriers impacting comprehensive educational supervision in schools in Sana’a, Yemen, a structured questionnaire was developed. The items in this instrument were derived from key barriers previously identified and validated through the Fuzzy Delphi Method. The questionnaire aimed to capture teachers’ perceptions of the supervisory challenges within their institutional contexts.

Step 1: Sample of the Study

A descriptive quantitative approach was employed, aligning with the study’s objective to assess systemic supervisory barriers from the teachers’ perspective. The study population comprised approximately 7,000 teachers working in schools that implemented comprehensive educational supervision in the capital city. Following Krejcie and Morgan’s sampling guidelines, a sample of 370 teachers was selected, surpassing the recommended minimum of 364 for a population of this size. This sample size ensured representativeness and allowed for statistical generalization to the broader population.

Step 2: Research Instrument Design

To gather the necessary data, a structured questionnaire was designed based on the study objectives, relevant literature, validated prior instruments, and the Fuzzy Delphi procedures outlined in Section 2.2. The instrument consisted of 11 items, each corresponding to a specific technical barrier affecting the effectiveness of comprehensive educational supervision. A five-point Likert scale was used to measure participants’ perceptions of each barrier, with response options ranging from “Very Low” (1 point) to “Very High” (5 points). The full scale included very low (1), low (2), moderate (3), high (4), and very high (5).

This structure enabled respondents to express their views clearly and allowed for the consistent quantification of data for analysis.

Step 3: Data Collection Procedures

The questionnaire was distributed across four educational districts in Amanat Al-asima Sana’a, where comprehensive educational supervision is currently being practiced. These districts were selected from among the ten official educational zones in the city of Amanat Al-Asima, Yemen.

Teachers from private schools that implemented comprehensive supervision practices were also included. Completed questionnaires were collected through school administrations and returned directly to the researcher.

*2.2.2 Application of the Fuzzy Set Theory to prioritize and rank the evaluated technical barriers*

In this step, the collected responses were converted into fuzzy numbers using the predefined scale (see Table 2), enabling the representation of uncertainty in respondents’ judgments. The average fuzzy rating for each barrier was calculated across all respondents using Equation 1. These fuzzy values were then defuzzified into crisp scores using the centroid (center of gravity) method, as described in Equation 2 (see Table 7).

**Table 7.** Respondents’ scores using a fuzzy rating scale.

Respondent No	B1			B2			… .	B11		
	*m1*	*m2*	*m3*	*m1*	*m2*	*m3*	*… .*	*m1*	*m2*	*m3*
**R1**	0	0	0.2	0.6	0.6	1	… .	0	0.2	0.4
**R2**	0	0.2	0.4	0	0.6	0.4	… .	0	0.2	0.4
**R3**	0.6	0.8	1	0.6	0.8	1	… .	0.2	0.4	0.6
**R4**	0.2	0.4	0.6	0.2	0.4	0.6	… .	0	0	0.2
**R5**	0	0	0.2	0.4	0.4	0.8	… .	0	0	0.2
**R6**	0.4	0.6	0.8	0	0.2	0.4	… .	0.4	0.6	0.8
**R7**	0.2	0.4	0.6	0.4	0.4	0.8	… .	0.6	0.8	1
**R8**	0.4	0.6	0.8	0.4	0.4	0.8	… .	0.4	0.6	0.8
**R9**	0.2	0.4	0.6	0.2	0.4	0.6	… .	0.2	0.4	0.6
**R10**	0.2	0.4	0.6	0.2	0.6	0.6	… .	0.2	0.4	0.6
**R11**	0.2	0.4	0.6	0.2	0.4	0.6	… .	0.4	0.6	0.8
**R12**	0.4	0.6	0.8	0.4	0.4	0.8	… .	0.4	0.6	0.8
**R13**	0	0	0.2	0	0	0.2	… .	0	0	0.2
**… ..**	… ..	… ..	… ..	… ..	… ..	… ..	… ..	… ..	… ..	… ..
**R369**	0.4	0.6	0.8	0.4	0.2	0.8	… .	0.4	0.6	0.8
**R370**	0.6	0.8	1	0.6	0.6	1	… .	0.6	0.8	1
**AFR**	0.235	0.415	0.615	0.257	0.421	0.648	… .	0.256	0.426	0.626
**CS**	0.422			0.442			… .	0.436		

Table presents the fuzzy scores of the 370 teacher respondents regarding the 11 technical barriers to effective educational supervision. Each response, recorded on a five-point Likert scale, was converted into triangular fuzzy numbers (m1, m2, m3). The table also includes the average fuzzy rating (AFR) and the resulting crisp scores (CS) for each barrier, enabling the prioritization for subsequent analysis.

Following the evaluation of the barriers and the computation of the average score for each based on respondents’ input, the aggregated scores were used to rank and categorize the barriers according to their impact on the effectiveness of comprehensive educational supervision.

*2.2.3 Application of the K-means clustering method to cluster the evaluated technical barriers*

The aim of the clustering process was to categorize the identified barriers into distinct groups based on their combined impact. This approach allows for a deeper understanding of the relative effects of these factors on the success of comprehensive educational supervision and enables the targeted prioritization of interventions. Clustering is a fundamental unsupervised machine learning technique that organizes unlabeled data into distinct clusters based on inherent similarities, such as proximity or density metrics. ^31– 34^

Unlike supervised learning, which relies on labeled outputs for training, clustering reveals hidden patterns without predefined categories, making it essential for exploratory analysis. By uncovering latent structures, clustering aids in hypothesis generation and provides valuable insights into strategic decision-making. ^70^ Among the various clustering techniques, K-means is particularly favored for its simplicity, computational efficiency, and versatility across disciplines. ^31, 32^

The algorithm partitions n data points into k clusters by minimizing the variance within each cluster. It follows an iterative process ^31– 35^: (1) randomly initializing k centroids; (2) assigning each data point to the nearest centroid; (3) recalculating centroids based on updated cluster memberships; and (4) repeating these steps until the model converges or reaches a predetermined iteration limit.

**Figure 2.**
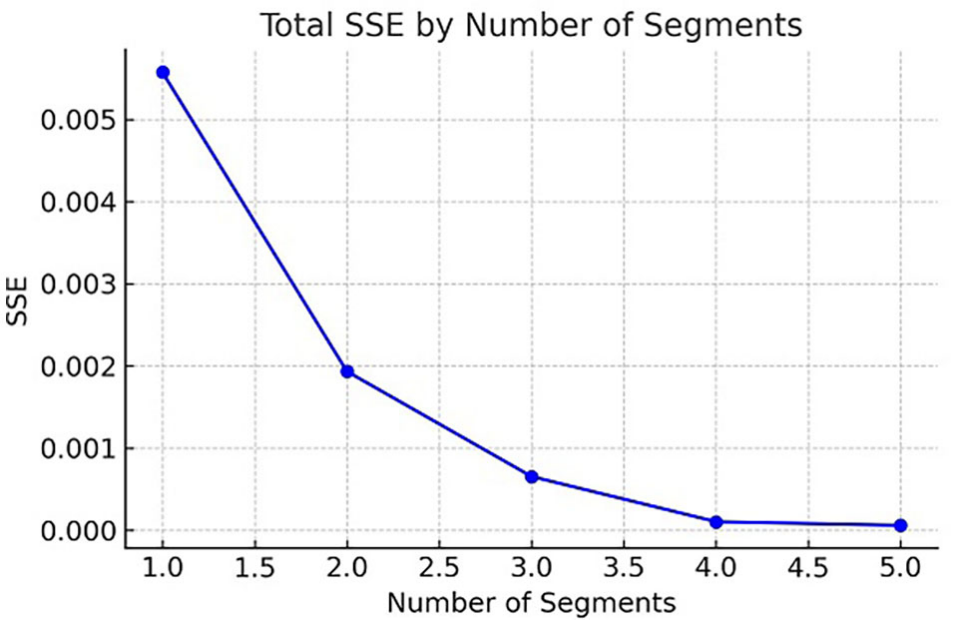
Elbow Method results for determining the optimal number of clusters. The plot illustrates the correlation between the within-cluster sum of squares (WCSS) and the number of clusters (k).

To determine the optimal number of clusters, the Elbow Method was employed using the Total Sum of Squared Errors (SSE) as a performance metric (see Figure 2). The SSE was computed for k values ranging from one to five. A significant reduction in SSE was observed between k = 1 (0.0055724) and k = 3 (0.0006534), indicating an improved model fit. Beyond k = 3, further reductions in SSE were minimal, suggesting diminishing returns. The inflection point at k = 3, known as the “elbow,” indicates an optimal balance between simplicity and explanatory power. Consequently, k = 3 was selected as the ideal number of clusters.

The final clustering model classified the barriers into a three-tier system based on their aggregated impacts. Segment 3 represents a high-priority danger zone, encompassing barriers with high average ratings from respondents, indicating significant concerns and severity. Segment 2 reflects a moderate level of concern, where the perceived impact of the barriers is notable but less critical. Segment 1 comprises low-priority or minimal-risk barriers, characterized by relatively low average ratings and limited influence on the effectiveness of comprehensive education supervision. This structured grouping enhances the interpretability of the results and provides a practical framework for prioritizing interventions and developing targeted policy responses.

### 2.4 Ethical approval and consent to participate

This study adhered to the principles of the Declaration of Helsinki and the national guidelines for research involving human participants. Official permission to conduct the study was secured from the Office of Education, Bani-Alhareth, Amanat Al Asimah, Republic of Yemen (Approval Reference No.: [72-9-24), authorizing data collection from participating schools. Participants received clear information about the study’s objectives and procedures and were assured that their participation was entirely voluntary, with the right to withdraw at any time, without consequences. Written informed consent was obtained from all participants before data collection, and no minors were included in this study.

## 3. Results

### 3.1 Fuzzy delphi method: Domain acceptability and expert agreement

The evaluation of domain acceptability aimed to assess the consistency of expert responses across all the identified barriers. The process began by calculating the distance between each expert’s fuzzy rating and the aggregated fuzzy score for each barrier. This deviation indicates how closely an individual expert’s judgment aligns with the group’s average perception. The mean of these deviations for each barrier was then computed to establish the threshold value. The results in Table 5 show that all 11 barriers had threshold values below the established cutoff of 0.2, ranging from 0.000 (for B9) to 0.150 (for B3 and B5). These findings suggest a high level of agreement among the experts for each variable. Furthermore, the domain-level threshold, derived by averaging the individual barrier thresholds, was calculated as 0.093, which is well below the acceptable upper limit. This confirmed the internal consistency of the expert evaluations and validated the overall domain of barriers for further analysis using the Fuzzy Delphi Method. Following the confirmation of domain validity, the focus shifted to assessing the expert consensus. This was achieved by identifying the number of experts whose ratings deviated from the group average by no more than 0.2 for each barrier. The percentage of experts who met this condition was calculated. The results in Table 6 reveal that all 11 barriers met or exceeded the minimum consensus threshold of 75%, with agreement levels ranging from 81% to 100%. Specifically, barriers B3, B5, B6, and B9 achieved full consensus (100%), while the lowest consensus rate was 81% for barriers B1, B2, B4, B7, and B10. The overall average expert agreement across all barriers was 89.77%, indicating a robust level of shared perceptions among the panel. Based on these results, all 11 barriers were accepted and retained for the final prioritization phase using defuzzified crisp scores.

In the final stage of the fuzzy Delphi analysis, the validated barriers were assessed and ranked according to their defuzzified crisp scores ( Table 8). These scores reflect the central tendency of expert judgments converted from fuzzy numbers into precise quantitative values using the centroid method. A threshold score of 0.4 was established to filter out low-priority barriers; any variable scoring below this cutoff was omitted from the final list. The results indicated that all 11 barriers achieved crisp scores well above the minimum threshold, underscoring their significance in the context of educational supervision challenges in Yemen’s medical schools. Among them, the barrier “insufficient availability of learning resources in schools” (B9) was ranked highest, with a crisp score of 0.800, followed by “inadequate financial allocations for supervision” (B8) at 0.788, and both “weak competencies of educational supervisors” (B4) and “lack of access to modern technologies” (B7) tied with a score of 0.763. The lowest-ranking yet accepted variables included “lack of dedicated offices for educational supervision” (B11) and “absence of training and rehabilitation halls” (B10), scoring 0.575 and 0.563, respectively. This ranking provides a clear prioritization of the most pressing barriers and serves as a critical reference point for targeted policy interventions and resource allocation to enhance the effectiveness of comprehensive educational supervision.

**Table 8.** Defuzzified crisp scores and ranking of educational supervision barriers based on expert evaluation.

Barrier	The average fuzzy rating for each variable	Rank	Decision
**B1**	0.638	9	Accepted
**B2**	0.758	5	Accepted
**B3**	0.688	8	Accepted
**B4**	0.763	3	Accepted
**B5**	0.713	7	Accepted
**B6**	0.725	6	Accepted
**B7**	0.763	3	Accepted
**B8**	0.788	2	Accepted
**B9**	0.800	1	Accepted
**B10**	0.563	11	Accepted
**B11**	0.575	10	Accepted

Table presents the defuzzified crisp scores and rankings of the 11 validated barriers to effective educational supervision evaluated by a panel of experts. Each barrier's crisp score was calculated using the centroid method, and the variables were ranked accordingly. A threshold score of 0.4 was used to determine inclusion; all barriers scored above this cutoff and were accepted for further consideration.

### 3.2 Assessment, ranking and clustering of technical supervision barriers

The sample included a diverse group of educators varying in gender, academic qualifications, years of experience, and school type. Specifically, 30% of the respondents (n = 111) were male and 70% (n = 259) were female. Regarding educational attainment, 40.8% (n = 151) held a high school diploma, 57.8% (n = 214) had a bachelor’s degree, and 1.4% (n = 5) had a master’s diploma or higher. In terms of professional experience, 32.4% (n = 120) had 1–5 years of teaching experience, 24.1% (n = 89) had 6–10 years, and 43.5% (n = 161) had over 10 years.

The questionnaire was distributed almost evenly across school types, with 51.4% of respondents (n = 190) from public schools and 48.6% (n = 180) from private ones. These demographic characteristics ensured that the collected data reflected a broad range of perspectives, enhancing the credibility and depth of the subsequent analysis of the perceived technical barriers.

The demographic data of the sample ( Table 9) indicate a predominantly female teaching workforce, with most holding at least a bachelor’s degree. A significant portion of respondents (43.5%) had more than ten years of teaching experience, suggesting that the dataset included insights from educators with substantial professional exposure. Additionally, the nearly equal distribution of respondents across public and private schools enhances the generalizability of the findings, allowing comparisons between different institutional contexts. Table 9 presents the full distribution of demographic variables among the sample:

**Table 9.** Demographic distribution of the study sample.

Variable	Category	Frequency	Percentage
**Gender**	Male	111	30.00%
	Female	259	70.00%
**Qualification**	High School	151	40.80%
	Bachelor’s Degree	214	57.80%
	Master’s Degree	5	1.40%
**Experience**	1–5 years	120	32.40%
	6–10 years	89	24.10%
	More than 10 years	161	43.50%
**School Type**	Public	190	51.40%
	Private	180	48.60%

Table summarizes the demographic distribution of the 370 teacher respondents who participated in the survey on technical barriers to educational supervision. The demographic information included gender, educational qualifications, years of teaching experience, and type of school. The data reflected a diverse and representative sample, supporting the reliability and generalizability of the study’s findings.

Table 10 presents the average fuzzy ratings for the various barriers impacting the efficacy of comprehensive educational supervision, along with their respective ranks. This evaluation, derived from the respondents’ input, offers a thorough overview of the challenges encountered in educational supervision. Table 11 illustrates the distribution of these barriers, which are categorized into three segments based on their perceived severity and impact. A total of 11 barriers were assessed, culminating in an overall sum of 100.0%. The table also includes the Sum of Squared Errors (SSE) for each segment. This distribution underscores the varying levels of concern associated with each segment, providing a clear overview of the challenges that require future attention. Segment 3, containing only one barrier, accounted for 9.1% of the total and reflected high-priority concerns perceived as critical threats to effective field supervision. In contrast, Segment 2 comprises seven barriers, representing 63.6% of the total, highlighting moderate-impact issues that significantly affect the supervision process of the internship. Segment 1 included three barriers, accounting for 27.3% of the total, indicating low-priority or minimal risk concerns that required less immediate attention. These barriers are characterized by relatively low average ratings and limited influence on the effectiveness of comprehensive educational supervision.

**Table 10.** Ranking of barriers impacting the effectiveness of educational supervision based on respondents’ input.

Barrier	The average fuzzy rating for each variable	Rank	Segment
B1	0.422	6	2
B2	0.442	2	2
B3	0.425	5	2
B4	0.467	1	3
B5	0.398	9	1
B6	0.440	3	2
B7	0.389	11	1
B8	0.391	10	1
B9	0.417	8	2
B10	0.421	7	2
B11	0.436	4	2

Table presents the average fuzzy ratings of the 11 technical barriers as perceived by the teacher respondents, along with their respective ranks and segmentations. The barriers were divided into three segments—high, moderate, and low priority—based on their perceived severity and impact on comprehensive educational supervision.

**Table 11.** Distribution and segmentation of barriers impacting educational supervision.

Segment	Number of barriers	Percentage %	SSE/Segment
**Segment 1**	3	27.3%	0.000047
**Segment 2**	7	63.6%	0.000607
**Segment 3**	1	9.1%	0.000000
**TOTAL**	11	100.0%	0.00065

Table details the distribution of the 11 educational supervision barriers across these three segments, indicating the percentage representation and Sum of Squared Errors (SSE) for each segment. The segmentation reflected varying levels of concern, with Segment 3 identifying the highest-priority barrier and Segment 1 containing those with minimal perceived impact.

## 4. Discussion

The Fuzzy Delphi Method (FDM) process confirmed the appropriateness and relevance of all 11 barriers to effective educational supervision. Each barrier met the necessary consensus and threshold criteria, demonstrating that the selected variables were not only theoretically grounded in the literature but also validated by expert opinion as reflective of the real-world challenges facing Yemen’s supervision system.

The high level of consensus among experts (average agreement of 89.77%) and a low domain-level threshold (0.093) indicate a consistent and strong agreement across the expert panel. These outcomes enhance the methodological rigor and credibility of barrier selection processes. Notably, the high crisp scores obtained for several barriers, particularly those related to resource limitations (e.g., insufficient learning materials and inadequate financial support), highlight their practical significance. These challenges are not just perceived concerns but are deeply embedded in the operational dynamics of the educational supervision system. Even barriers with relatively lower average ratings, such as the lack of training halls or dedicated office space, were retained in the final model, emphasizing their contextual importance despite being perceived as less urgent issues. The use of FDM in this study served as an effective filtering and validation mechanism, ensuring that only the most contextually relevant and expert-validated barriers were included in subsequent analytical phases. This is especially critical in complex decision-making environments, where aligning empirical evidence with expert insights enhances the focus, reliability, and applicability of advanced evaluation models, such as MCDM techniques or policy simulations. By incorporating expert judgment under conditions of uncertainty and applying structured fuzzy logic principles, the method strengthens the foundation for deeper investigation and prioritization. ^41, 44, 55^

Following FDM validation, ranking and clustering analyses ( Tables 9 and 10) provided further insights into how these barriers vary in severity and impact. Segmentation into three groups enabled the strategic categorization of challenges, offering a clearer roadmap for targeted interventions and policy reform.

The analysis identified a critical barrier in educational supervision: the weak competencies of educational supervisors (B4). This barrier, despite representing only 9.1% of total concerns, received the highest average score (0.467) and ranked first among all barriers, emphasizing its importance. Consistent with the existing literature, ^71– 73^ this finding highlights the vital role of competent supervision in teacher development, instructional improvement, and overall school enhancement. Competent supervisors possess essential skills in instructional leadership, communication, observation, feedback delivery, and the ability to analyze teaching practices and design appropriate interventions. ^15, 16^ Weak supervisory competencies can undermine the entire supervisory process, leading to unclear guidance, ineffective support, and hindered teacher development, ^74^ potentially compromising educational quality and impeding systemic improvement. This analysis emphasizes the need for immediate and sustained action to enhance supervisory competencies, as they form the foundation of effective educational supervision and improve teaching and learning outcomes. ^16– 18^ To address this critical issue, policymakers and educational institutions must prioritize comprehensive professional development programs for supervisors, focusing on continuous training, mentorship programs, tailored performance evaluation systems, and the development of instructional leadership capabilities. ^15, 17, 18, 72, 73^ Strengthening supervisory competencies is likely to have far-reaching effects, potentially mitigating the interconnected barriers within the educational system.

Furthermore, segment 2, comprising seven barriers and representing the largest portion (63.6%), included various moderate-impact issues influencing daily supervision operations. The most significant barrier, B2: Limited capability in creating effective supervisory plans (average score: 0.442), highlights the necessity of enhanced strategic planning skills among supervisors. This aligns with the understanding that well-developed supervisory plans are essential for effective supervision, providing structure, clear goals, and direction. ^58, 59^ Effective supervisory plans ensure a systematic rather than a reactive process, aligning supervision with instructional improvement. These plans must be goal-oriented, realistic, adaptable, time-bound, and developed collaboratively with the teachers. The ability to create and implement strong supervisory plans is central to ensuring purposeful and focused supervision aligned with educational goals. ^13, 14^ Conversely, poorly planned supervision can be inconsistent or unclear, limiting its impact on teacher development and student learning. Equally concerning is B6: Poor relationships between supervisors and teachers (0.440), which points to underlying communication and trust issues. Effective supervision relies not only on technical competence but also on interpersonal relationships that foster a supportive and collaborative school environment. A professional relationship built on trust and communication encourages teacher openness, responsiveness to feedback, and collaboration. ^18, 64^

In contrast, weak relationships or lack of trust may result in resistance and reduce the impact of supervisory efforts. Additional barriers in this segment include B11: Lack of dedicated offices for educational supervision (0.436) and B3: Limited capability in implementing training programs (0.425). These barriers indicate both infrastructural deficiencies and limited pedagogical leadership, suggesting a need for resource investment and capacity building. Well-designed and timely training programs directly enhance teachers’ instructional skills, promote consistency in instructional quality, and foster a culture of professional growth. ^10, 11^ The distribution of barriers in Segment 2 underscores the need for a comprehensive improvement strategy that addresses both relational and structural challenges. Enhancing supervisors’ abilities across three key dimensions—planning, relational engagement, and training delivery—is foundational to improving the overall effectiveness of supervision systems.

This approach would involve the socioeconomic status: Strengthening planning capabilities to improve the quality, consistency, and outcomes of the educational supervision process; developing meaningful relationships between supervisors and teachers to create a supportive and collaborative environment is essential; improving the design and implementation of impactful training programs to enhance teacher performance and professional development is crucial.

By addressing these interconnected aspects, supervisors can provide more meaningful feedback, manage their time effectively, and identify key areas for improvement. ^13^ This comprehensive approach would significantly enhance the quality and impact of educational supervision, ultimately benefiting the development of teachers and student learning outcomes.

Finally, segment 1, comprising three barriers and representing 27.3% of the total, included concerns that were considered low-priority or minimal risk. These barriers are marked by relatively low average ratings and limited impact on the effectiveness of comprehensive educational supervision. Despite being rated as lower priority, the barriers in Segment 1 highlight structural limitations that could negatively affect the supervision process if not addressed. For example, limited teacher acceptance of modern supervisory methods can stifle innovation and diminish the effectiveness of such interventions. When teachers resist collaborative evaluation, peer feedback, or data-informed coaching, it undermines the culture of trust and continuous improvement that is essential for effective supervision. ^22^ Similarly, inadequate financial allocations for supervision activities restrict supervisors’ ability to conduct school visits, attend professional development programs, or acquire necessary materials, resulting in poorly planned and under-resourced supervision systems. ^8, 23^ Additionally, the lack of access to modern technologies limits the use of digital tools for classroom observation, feedback, training and communication. Without these tools, supervision becomes less interactive, data-informed, and responsive. ^19, 65^ While these barriers may not have the most immediate impact, their cumulative effect over time can impede broader reform efforts, stifle innovation, and exacerbate other challenges in educational supervision. Therefore, continuous monitoring and proactive mitigation of lower-ranked issues are crucial for sustaining long-term improvements.

The low average ratings and limited influence of these barriers indicate that resources and efforts should primarily focus on addressing more critical issues in comprehensive educational supervision. Nevertheless, it remains important to monitor these low-priority barriers, as they could potentially affect the overall effectiveness of supervision if neglected for an extended period.

In summary, the integrated FDM and clustering analysis offered a multilayered understanding of the barriers hindering educational supervision in Yemen. The findings emphasize the urgency of enhancing supervisory competencies and highlight the need to address moderate-impact barriers related to planning, relationships, infrastructure, and training. Low-priority issues should not be ignored, as their future impact may become more significant in a changing educational landscape. Collectively, these insights provide a strategic framework for policymakers, educational leaders, and stakeholders to allocate resources, design interventions, and improve supervisory effectiveness, ultimately contributing to more robust and equitable educational outcomes.

## 5. Conclusion

This study systematically investigated the technical barriers undermining the effectiveness of comprehensive educational supervision in Amanat Al Asimah, Yemen, using a robust mixed-methods framework. Eleven key challenges were identified, validated through the Fuzzy Delphi Method, and prioritized using fuzzy set theory and K-means clustering. The Fuzzy Delphi results demonstrated strong expert consensus, with a domain-level threshold of 0.093 and an average agreement of 89.77%, affirming the contextual relevance of the selected barriers. Through clustering analysis, the barriers were segmented into three priority levels. Segment 3, comprising a single high-priority issue—weak supervisory competencies—emerged as the most critical concern, underscoring the central role of professional capacity in enabling effective supervision. Segment 2 contained seven moderate-priority barriers, including planning deficiencies, strained supervisor–teacher relationships, and inadequate training infrastructure. Segment 1 grouped three low-priority barriers, such as resistance to modern supervision methods and technological or financial constraints, which, though less urgent, could accumulate into significant challenges if left unaddressed.

These findings carry important implications for policymakers, educational leaders, and planners operating in fragile and resource-limited contexts. The study offers a data-driven roadmap for prioritizing reforms, emphasizing the urgent need to invest in supervisory training, institutional planning, and resource allocation. The segmentation of barriers provides a practical framework for designing targeted, phased interventions. Additionally, the approach used in this study can be replicated or adapted to other conflict-affected or low-income settings, contributing to broader efforts in educational reform and capacity building.

However, the study also faces several limitations. It was geographically restricted to Amanat Al Asimah, potentially limiting generalizability across Yemen’s diverse regions. Furthermore, it focused primarily on teachers’ and supervisors’ perspectives, excluding voices from students, principals, and policymakers. Also, it concentrated on technical barriers, leaving out sociopolitical or cultural dimensions that may influence supervisory effectiveness. Future research should expand geographically, incorporate multiple stakeholders, and explore non-technical factors influencing educational supervision. Longitudinal and experimental designs could also evaluate how barriers evolve and how proposed solutions perform over time. Comparative studies across similar contexts, or using advanced analytical techniques, such as neural network analysis, ^75^ could provide valuable benchmarks and inform regional or global supervisory strategies.

In summary, this study highlights the urgency of strengthening supervisory competencies and institutional frameworks to enhance the quality and effectiveness of educational supervision in Yemen. By integrating expert validation, teacher feedback, and advanced clustering techniques, it presents a scalable and evidence-based framework that can inform strategic planning, policy development, and educational resilience in post-crisis and low-resource environments.

## Ethics and consent

This study adhered to the principles of the Declaration of Helsinki and the national guidelines for research involving human participants. Official permission to conduct the study was secured from the Office of Education, Bani-Alhareth, Amanat Al Asimah, Republic of Yemen (Approval Reference No.: 72-9-24), authorizing data collection from participating schools. Participants received clear information about the study’s objectives and procedures and were assured that their participation was entirely voluntary, with the right to withdraw at any time, without consequences. Written informed consent was obtained from all participants before data collection, and no minors were included in this study.

## Declaration of generative AI and AI-assisted technologies in the writing process

During the preparation of this work the author(s) used [DeepSeek v3, Paperpal, Quillbot, and ChatGPT] for language refinement and structure. After using this tools, the author(s) reviewed and edited the content as needed and take(s) full responsibility for the content of the publication.

## Data Availability

Not applicable. ***Figshare:*** Toward Effective Educational Supervision in Yemen: A Hybrid Fuzzy Delphi and Clustering Analysis of Technical Barriers. Doi: https://doi.org/10.6084/m9.figshare.29483738.v4. ^76^ ***The project contains the following underlying data:***
-
**Supplementary material – Toward Effective Educational Supervision in Yemen.xlsx.** All data, and processing results related to this study are presented in this file. This file integrates the application of the Fuzzy Delphi method to assess the consensus and suitability of the items, validated by 16 experienced educational experts. It also contains the collected and processed data from a quantitative survey targeting 370 teachers, along with the Fuzzy Set Theory processes for data aggregation. Additionally, it illustrates the K-means clustering results for segmenting the barriers based on their impacts. All this data is merged into a single Excel-based tool. This source also includes the values behind the results reported. This file also includes the values behind the measures reported in all analysis and discussion sections, as well as the values used to construct tables and figures. This supplementary resource also provides detailed support for replicating the study’s methods and results.
-
**Supplementary files (copy of Ethical approval, Consent Form for participants and Questionnaires for data collection) (Original Arabic Version)**
-
**Supplementary Files – English Translation of Original Documents** This data are publicly available and can be accessed through the following repository ( https://figshare.com/articles/dataset/Supplementary_material_-_Toward_Effective_Educational_Supervision_in_Yemen-A_Hybrid_Fuzzy_Delphi_ and_Clustering_Analysis_of_Technical_Barriers/29483738?file=56000291) and archived via [ https://doi.org/10.6084/m9.figshare.29483738.v4] ^76^. Data are available under the terms of the Creative Commons Attribution 4.0 International license (CC-BY 4.0).
