# Supplementary material for: Toward Effective Educational Supervision in Yemen: A Hybrid Fuzzy Delphi and Clustering Analysis of Technical Barriers
Source: F1000Res. 2026 Feb 9;14:956. Originally published 2025 Sep 22. [Version 2] doi: 10.12688/f1000research.167498.2 (PMC12905534; doi:10.12688/f1000research.167498.2)
Supplement: Peer review history

# Reviewer report: Reviewer response for version 2

**DOI:** 10.5256/f1000research.195822.r456883

**Published:** 2026-02-13

**Authors:** Indra Prasetia

**Affiliations:** 1 Universitas Muhammadiyah Sumatera Utara, Medan, Indonesia

I have read the proposed revisions to the manuscript, and it has undergone significant improvements as I suggested.

 My decision: The manuscript is acceptable for indexing.

Is the work clearly and accurately presented and does it cite the current literature?

Partly

If applicable, is the statistical analysis and its interpretation appropriate?

Yes

Are all the source data underlying the results available to ensure full reproducibility?

Yes

Is the study design appropriate and is the work technically sound?

Partly

Are the conclusions drawn adequately supported by the results?

Yes

Are sufficient details of methods and analysis provided to allow replication by others?

Partly

Reviewer Expertise:

education (education manajemen and leadership)

I confirm that I have read this submission and believe that I have an appropriate level of expertise to confirm that it is of an acceptable scientific standard.

# Reviewer report: Reviewer response for version 2

**DOI:** 10.5256/f1000research.195822.r456884

**Published:** 2026-02-10

**Authors:** Salah AL-Hagree

**Affiliations:** 1 Department of Computer Sciences, Sana'a University, Sanaa, Yemen

I have no further comments to make.

Is the work clearly and accurately presented and does it cite the current literature?

Yes

If applicable, is the statistical analysis and its interpretation appropriate?

Yes

Are all the source data underlying the results available to ensure full reproducibility?

Yes

Is the study design appropriate and is the work technically sound?

Yes

Are the conclusions drawn adequately supported by the results?

Yes

Are sufficient details of methods and analysis provided to allow replication by others?

Yes

Reviewer Expertise:

Natural Language Processing, Data Science, Machine Learning, Deep Learning, and  Artificial Neural Networks

I confirm that I have read this submission and believe that I have an appropriate level of expertise to confirm that it is of an acceptable scientific standard.

# Reviewer report: Reviewer response for version 1

**DOI:** 10.5256/f1000research.184615.r435827

**Published:** 2026-01-20

**Authors:** Salah AL-Hagree

**Affiliations:** 1 Department of Computer Sciences, Sana'a University, Sanaa, Yemen

Correcting the values ​​in Table 5,  Percentage of Experts (%) or Average 90% ( **Average 89.73 **)

  - Percentage of Experts, d ≤ 0.2    81.25%    81.25%    100.00%    81.25%    100.00%    100.00%    81.25%    93.75%    100.00%    81.25%    87.50%

  - Percentage of Experts, d ≤ 0.2    81%    81%    100%    81%    100%    100%    81%    94%    100%    81%    88%

 Based on the previous correction, the following is corrected:

  3. Results

  The overall average expert agreement across all barriers was 90%, 

  4. Discussion

  The high level of consensus among experts (average agreement of 90%) 

  5. Conclusion

 - and an average agreement of 90%, affirming the contextual relevance of the selected barriers.

 - Future research should expand the use of other classification techniques, such as the technique in the reference: https://zenodo.org/records/17251460

 -The method should clearly incorporate the concept of **Hybrid**: A **Hybrid Fuzzy Delphi **and Clustering Analysis of Technical Barriers

 - References related to Yemen

 1- Alqasemi, F., Salah, A. H., Aqlan, A., Alalayah, K. M., Almotwakl, Z., & Hadwan, M. (2021, March). Education Data Mining For **Yemen **Regions Based On Hierarchical Clustering Analysis. In 2021 International Conference of Technology, Science and Administration (ICTSA) (pp. 1-4). IEEE. https://doi.org/10.1109/ICTSA52017.2021.9406544‏

 2- Al, S., & Alwan, S. M. (2025). Neural Network Analysis of Factors Influencing Forgotten Financial Remittances in **Yemen**. Journal of Economic Cooperation and Development, 46(2), 71-102. https://doi.org/10.5281/zenodo.17251460‏

Is the work clearly and accurately presented and does it cite the current literature?

Yes

If applicable, is the statistical analysis and its interpretation appropriate?

Yes

Are all the source data underlying the results available to ensure full reproducibility?

Yes

Is the study design appropriate and is the work technically sound?

Yes

Are the conclusions drawn adequately supported by the results?

Yes

Are sufficient details of methods and analysis provided to allow replication by others?

Yes

Reviewer Expertise:

Natural Language Processing, Data Science, Machine Learning, Deep Learning, and  Artificial Neural Networks

I confirm that I have read this submission and believe that I have an appropriate level of expertise to confirm that it is of an acceptable scientific standard, however I have significant reservations, as outlined above.

# Response

**Published:** 2026-01-26

**Authors:** Adel A. Nasser

**Affiliations:** Information Systems, Not Applicable, Sa'ada, Sa'ada, Yemen

**Responses to Peer Review Report of Assis. Prof.  Salah AL-Hagree, Department of Computer Sciences, Sana'a University, Sanaa, Yemen** 

**Dear Prof. Salah AL-Hagree,**

 Thank you for your thoughtful review and for the insightful comments provided on our manuscript. Your feedback has been invaluable in guiding our revisions.

**Comments 1,2:** Correcting the values ​​in Table 5,  Percentage of Experts (%) or Average 90% ( Average 89.73 )

  - Percentage of Experts, d ≤ 0.2    81.25%    81.25%    100.00%    81.25%    100.00%    100.00%    81.25%    93.75%    100.00%    81.25%    87.50%

  - Percentage of Experts, d ≤ 0.2    81%    81%    100%    81%    100%    100%    81%    94%    100%    81%    88%

**Response: **The authors acknowledge the reviewer's correct observation regarding the precision of the data in Table 5. The specified percentages for the row "Percentage of Experts, d ≤ 0.2" have been revised to two decimal places as suggested: 81.25%, 81.25%, 100.00%, 81.25%, 100.00%, 100.00%, 81.25%, 93.75%, 100.00%, 81.25%, 87.50%. The summary value in the text has been correspondingly updated to "Average 89.73%." We are grateful for this correction.

 .

**Comments 3-5: **Updating the referenced average value throughout the manuscript:

- Results section: “The overall average expert agreement across all barriers was 90%”,
- Discussion section: “The high level of consensus among experts (average agreement of 90%)
** **
- Conclusion - and an average agreement of 90%, affirming the contextual relevance of the selected barriers.

**Response:** We thank the reviewer for their consistency in pointing out the related text. Following the correction to the data in Table 5, we have also updated the referenced average value throughout the manuscript

- In the Results section, the sentence now reads: "The overall average expert agreement across all barriers was 89.73%."
- In the Discussion section, the text has been amended to: "The high level of consensus among experts (average agreement of 89.73%)..."
- In the Conclusion section, the text has been amended to: "and an average agreement of 89.77%, ...”
- In the Abstract section, the sentence now reads” and an average expert consensus of 89.77%, indicating strong agreement

**Comment 6**: - Future research should expand the use of other classification techniques, such as the technique in the reference: https://zenodo.org/records/17251460

**Response:** We appreciate this suggestion and will explore other classification techniques. The revised manuscript now incorporates a discussion of the suggested reference in the Literature Review/Future Work section.

**Comment 7**: The method should clearly incorporate the concept of Hybrid: A Hybrid Fuzzy Delphi and Clustering Analysis of Technical Barriers

**Response: Response:** Thank you for this insightful recommendation. We have explored this integration in the revised manuscript. The methodology section now clearly illustrates the two-tiered hybrid analytical framework that integrates methodological approaches for barrier validation and data-driven prioritization. The sequential steps of the framework are now explicitly described.

**Comment 8**: - References related to Yemen

 1- Alqasemi, F., Salah, A. H., Aqlan, A., Alalayah, K. M., Almotwakl, Z., & Hadwan, M. (2021, March). Education Data Mining For Yemen Regions Based On Hierarchical Clustering Analysis. In 2021 International Conference of Technology, Science and Administration (ICTSA) (pp. 1-4). IEEE. https://doi.org/10.1109/ICTSA52017.2021.9406544‏

 2- Al, S., & Alwan, S. M. (2025). Neural Network Analysis of Factors Influencing Forgotten Financial Remittances in Yemen. Journal of Economic Cooperation and Development, 46(2), 71-102. https://doi.org/10.5281/zenodo.17251460

**Response:** We acknowledge the importance of citing the relevant literature related to Yemen. We appreciate the references provided, and the revised manuscript now includes five new relevant references (cited as references 28, 29, 49, 54, and 74).

 We hope that our responses and the corresponding revisions adequately address your concerns and have improved our manuscript. Thank you again for your valuable time and insights

# Reviewer report: Reviewer response for version 1

**DOI:** 10.5256/f1000research.184615.r417161

**Published:** 2025-10-09

**Authors:** Indra Prasetia

**Affiliations:** 1 Universitas Muhammadiyah Sumatera Utara, Medan, Indonesia

1.    Is the work clearly and accurately presented and does it cite the current literature? 

 Comment: This work is presented clearly and accurately. However, there are outdated data reviews in uncovering research issues. (e.g. The education system, which once showed progress with enrollment rates increasing from 71.3% to 97.5% between 1999 and 2013, has since experienced significant setbacks. By the 2015/2016 academic year, around 1.8 million children were out of school, and over 1,100 schools had been either destroyed or repurposed for emergency use. ^27^). More updated data support is needed to reinforce current issues.

 2.    Is the study design appropriate and is the work technically sound? [Please consider whether appropriate controls have been included]

 Comment: This study uses an integrative approach to explore and prioritise obstacles in the context of education monitoring in Yemen. My analysis shows that this integrative approach generally refers to research using mixed methods. If this is a mixed design, it needs to be explained in detail with clear references (a diagram of the stages also needs to be provided). 

 3.    Are sufficient details of methods and analysis provided to allow replication by others? [Please consider whether the authors have adhered to relevant reporting guidelines]

 Comment: The methods and analysis have been sufficiently explained, but there are a few shortcomings. The stages of theory-based data analysis (literature review) have been applied in the research, and a number of important themes or variables such as B1, B2, … B11 have been identified. In my opinion, this is content analysis that produces themes (is my opinion correct?). If this is correct, then the literature data and analysis need to be presented, leading to the conclusion of the themes found (B1, B2...B11), so that these themes are supported by accurate data and analysis.

 4.    If applicable, is the statistical analysis and its interpretation appropriate? 

 Comment: Already in accordance

 5.    Are all the source data underlying the results available to ensure full reproducibility?

 [We require that all data underlying figures and tables are provided (unless there are issues of confidentiality) as part of the article or deposited in a public repository. The data should be described in a usable and accessible format]

 Comment: The data based on the literature reviewed in Section 2.1 needs to be presented. (I suggest presenting it in a simple table format.)

 6.    Are the conclusions drawn adequately supported by the results? 

 [Please consider whether any limitations are discussed in sufficient detail]

 Comment: Very adequate.

Is the work clearly and accurately presented and does it cite the current literature?

Partly

If applicable, is the statistical analysis and its interpretation appropriate?

Yes

Are all the source data underlying the results available to ensure full reproducibility?

Yes

Is the study design appropriate and is the work technically sound?

Partly

Are the conclusions drawn adequately supported by the results?

Yes

Are sufficient details of methods and analysis provided to allow replication by others?

Partly

Reviewer Expertise:

education (education manajemen and leadership)

I confirm that I have read this submission and believe that I have an appropriate level of expertise to confirm that it is of an acceptable scientific standard, however I have significant reservations, as outlined above.

# Response

**Published:** 2026-01-26

**Authors:** Adel A. Nasser

**Affiliations:** Information Systems, Not Applicable, Sa'ada, Sa'ada, Yemen

**Responses to Peer Review Report of Prof. Indra Prasetia, Universitas Muhammadiyah Sumatera Utara, Medan, Indonesia**

**Dear Prof. Indra Prasetia,**

 We sincerely thank the reviewers for their insightful comments and constructive feedback, which have greatly improved the quality and clarity of this manuscript. Below, we address each point in detail and describe the corresponding revisions made

**Comment 1:** This work is presented clearly and accurately. However, there are outdated data reviews in uncovering research issues. (e.g. The education system, which once showed progress with enrollment rates increasing from 71.3% to 97.5% between 1999 and 2013, has since experienced significant setbacks. By the 2015/2016 academic year, around 1.8 million children were out of school, and over 1,100 schools had been either destroyed or repurposed for emergency use.27). More updated data support is needed to reinforce current issues.

**Response:** Thank you for your valuable and constructive feedback regarding the use of outdated data in the introduction. We agree that current statistics are essential for accurately framing the research problem within Yemen's severe and ongoing humanitarian crisis. In direct response to your comment, we have thoroughly revised the introduction by connecting the outdated with the most current, authoritative data available from major UN and humanitarian organizations (2024-2025) (Two references were added [28, 29]).

**Comment 2:** This study uses an integrative approach to explore and priorities obstacles in the context of education monitoring in Yemen. My analysis shows that this integrative approach generally refers to research using mixed methods. If this is a mixed design, it needs to be explained in detail with clear references (a diagram of the stages also needs to be provided). 

**Response: **Thank you for your constructive feedback. In response, we have revised the Methodology section. New paragraphs describing the framework were added, a new diagram was provided, and the entire section was restructured to explain the detailed steps of the proposed framework in a clear sequence, supported by appropriate references.

**Comment 3:** The methods and analysis have been sufficiently explained, but there are a few shortcomings. The stages of theory-based data analysis (literature review) have been applied in the research, and a number of important themes or variables such as B1, B2, … B11 have been identified. In my opinion, this is content analysis that produces themes (is my opinion correct?). If this is correct, then the literature data and analysis need to be presented, leading to the conclusion of the themes found (B1, B2...B11), so that these themes are supported by accurate data and analysis.

**Response: **Thank you for your insightful observation. In response, we have revised the section to explicitly frame this methodological step. The 11 core barriers (B1–B11) are now clearly presented as direct outcomes of the analysis, with each theme succinctly defined and immediately supported by its foundational citations from the literature. This directly links the conclusions to the supporting data, ensuring the themes are evidence-based. To maintain a logical flow and avoid duplication, the detailed interpretation and synthesis of these findings are elaborated in the dedicated Discussion.

**Comment 4:** If applicable, is the statistical analysis and its interpretation appropriate? 

 Comment: Already in accordance

**Response: Thank you**

**Comment 5: **The data based on the literature reviewed in Section 2.1 needs to be presented. (I suggest presenting it in a simple table format.).

**Response: **Thank you for your insightful observation.  A new table (Table 1) has been added to provide a concise, at-a-glance summary of all identified barriers, reinforcing the output of the content analysis.

**Comment 6: **Are the conclusions drawn adequately supported by the results? 

 Comment: Very adequate.

**Response: Thank you.**

 We hope that these responses adequately address your concerns and further strengthen our manuscript. Thank you again for your valuable insights!
